# Utility and limitations of measures of health inequities: a theoretical perspective

**DOI:** 10.3402/gha.v8.27591

**Published:** 2015-09-09

**Authors:** Olakunle Alonge, David H. Peters

**Affiliations:** Department of International Health, Johns Hopkins Bloomberg School of Public Health, Baltimore, MD, USA

**Keywords:** health inequities, program evaluation, theory of social justice, measures of inequality

## Abstract

**Summary box:**

**What is already known on this subject?**

Various measures have been used in quantifying health inequities among populations in recent times; most of these measures were derived to capture the socioeconomic inequalities in health. These different measures do not always lend themselves to common interpretation by policy makers and health managers because they each reflect limited aspects of the concept of health inequities.

**What does this study add?**

To inform a more appropriate application of the different measures currently used in quantifying health inequities, this article explicates common theories underlying the definition of health inequities and uses this understanding to show the utility and limitations of these different measures. It also suggests some key features of an ideal indicator based on the conceptual understanding, with the hope of influencing future efforts in developing more robust measures of health inequities. The article also provides a conceptual ‘product label’ for the common measures of health inequities to guide users and ‘consumers’ in making more robust inferences and conclusions.

This paper examines common approaches for quantifying health inequities and assesses the extent to which they incorporate key theories necessary for explicating the definition of health inequity. The first theoretical analysis examined the distinction between inter-individual and inter-group health inequalities as measures of health inequities. The second analysis considered the notion of fairness in health inequalities from different philosophical perspectives. To understand the extent to which different measures of health inequities incorporate these theoretical explanations, four criteria were used to assess each measure: 1) Does the indicator demonstrate inter-group or inter-individual health inequalities or both; 2) Does it reflect health inequalities in relation to socioeconomic position; 3) Is it sensitive to the absolute transfer of health (outcomes, services, or both) or income/wealth between groups; 4) Could it be used to capture inequalities in relation to other population groupings (other than socioeconomic status)? The measures assessed include: before and after measures within only the disadvantaged population, range, Gini coefficient, Pseudo-Gini coefficient, index of dissimilarity, concentration index, slope and relative indices of inequality, and regression techniques. None of these measures satisfied all the four criteria, except the range. Whereas each measure quantifies a different perspective in health inequities, using a measure within only the disadvantaged population does not measure health inequities in a meaningful way, even using before and after changes. For a more complete assessment of how programs affect health inequities, it may be useful to use more than one measure.

Health inequalities are inequalities in health determinants and outcomes between individuals or groups. The term health disparity has been used interchangeable with health inequality, especially in the United States (1). Health inequalities at the global level have raised much concern (2). Within-country health inequalities have however attracted less attention in low- and middle-income countries (LMIC) (3). This lack of attention may be attributable to LMIC stakeholders’ focus on achieving the health-related Millennium Development Goals (MDGs), which are measured at the aggregate level (3). With the 2015 deadline for achieving the MDGs already here, there have been calls for a post-2015 framework that tracks within-country inequalities and prescribes goals for reducing or eliminating them (3).

Indeed, the post-2015 agenda or Sustainable Development Goal, as it relates to health, is based on the central notion of addressing health inequalities in all countries: by promoting universal health coverage for people of all ages based on a life course model (3). The life course approach is one of several models that have been used in explaining the etiologies of health inequalities; others include the cultural–behavioral, materialist, and psychosocial models (Box 1) (4–6). Irrespective of the causal model assumed for explaining health inequalities however, there is a consensus that inequalities are not self-correcting, but they require interventions (policies and programs) to change (4, 5).

***Box 1*. Examples of models for explaining the etiologies of health inequalities**

1. *Life course model:* Health inequalities occurring at any age are results of differences in the accumulation of behavioral, psychosocial, material, and other risk factors for ill-health at all preceding life stages or critical periods prior to the current age (5, 6).
2. *Cultural-behavioral model:* Health inequalities are results of differences in behavioral or lifestyle choices which consequently lead to ill-health and these choices are influenced by cultural norms and other environmental factors (5, 6).
3. *Psychosocial model:* Health inequalities are due to differences in psychological stress from factors such as less control at work, less job security, lower levels of social support, and living in communities with higher crime. Such psychological stress leads directly to ill-health or indirectly through its effect on behaviors/lifestyle (5, 6).
4. *Materialist model:* Health inequalities are due to differences in exposure to material factors outside of one's control, e.g. housing and hazard in workplace, which affect health and are related to differences in position within a social structure (4, 5).

To be sure, not all health inequalities are readily amenable to interventions, (7–9) but so far as some are attributable to avoidable disparities in the distribution of social determinants of health – such as income, education, and access to health services – this provides a moral reason, and in most societies an emotionally compelling reason, to institute mitigating policies (7–9). Accordingly, health inequities have been distinguished as a subset within the broader category of health inequalities: various definitions characterize health inequities as unfair and/or avoidable health inequalities (8–10).^1^ Even bracketing moral concerns, there are other well-documented justifications for interventions to address health inequities thus understood, including the facts that they hamper aggregate economic development, contribute to the spread of ill-health, and undermine societal stability that are critical for human development (11–13).

Different measures have been used in the past to quantify the effect of public health interventions on health inequities. However, not all of these measures reflect the conceptual underpinnings of the definition of health inequities. Indeed, some programs that have been suggested as effective in addressing health inequities may not hold true once some defining constructs are incorporated into the measure of health inequities used in evaluating these programs. This article sets forth four theoretical analyses to understand how to operationalize the definition of health inequities for evaluation of public health programs. The first two arguments examine the conceptual basis for defining health inequalities as inter-individual or inter-group differences and qualified health inequities as unfair health inequalities from different philosophical perspectives. The last two analyses examine common approaches for assessing health inequities and their utility for program evaluation in the light of the definition set forth in the previous arguments.

## Defining health inequities: between inter-individual and inter-group differences

Black et al. (14) in their famous report titled ‘Inequalities in Health; Report of a Research Working Group’ demonstrated that inequities in mortality between social classes in England and Wales had increased steadily from 1955 to 1972. Le Grand and Rabin (15) using an inter-individual approach (that looked at individual variation in average lifespan without any reference to social class) contended that contrary to the Black report, health inequities in Britain had in fact reduced over the same period. In the recent decades, a similar debate had ensued between Murray et al. (16, 17) and Braveman et a. (18). Murray et al. (16) in an effort to provide a basis for cross-country comparative analysis of health determinants had defined health inequities on the basis of inter-individual inequalities in health within a population without any particular reference to social groupings and had opined that this approach could be used to guide evaluation of public health interventions. Braveman et al. (18) however countered that such proposal represents a misconception of the measure of health inequities. They argued that the definition of health inequities should be based on a moral conception of inter-group differences in health and its determinants and only such definition should guide interventions for improving population health (18).

At the heart of these arguments are the fundamental difficulties inherent in defining and operationalizing the definitions of health inequities (19). The operationalization of health inequities on the basis of inter-individual differences in health borrows heavily from economic theories of income inequality which conceptualize income as being normally distributed and measured on a ratio scale (20). This operationalization is however difficult to achieve with valuation of health states. Indeed, health cannot be measured on a ratio scale due to its multidimensionality (19). For instance, can we say that the absolute difference between two health states, x and y, is the same as that between two other health states, p and q, in terms of both magnitude and direction? To circumvent this issue, biomarkers (such as body mass index and height for age) and measures of life or health expectancies have sometimes been used as unidimensional proxies of health state in assessing inter-individual differences.

For one measure of inter-individual differences certain mathematical parameters were proposed to approximate the distribution of health expectancies with that of income on a ratio scale (21). This measure called ‘inter-individual differences’ compares each individual's health to every other individual's health (21). Whereas proponents of this approach duly noted that the measure uses a different weight for health differentials at the end of age distributions and does not exclude health differentials that are due to unavoidable factors (e.g. genes). They argued that such distinctions are not important for policy actions since future health technologies can significantly alter our judgment of factors that we classify as unavoidable (21).

The notion of inter-group differences for defining health inequities is based on the theory of social justice and it emphasizes improving outcomes for the worst-off group to approach the state among the well-off group as much as possible (22, 23). Sen (19) pointed out that such measurement of health requires only an ordinal comparison of health states between social groups and does not require any strong assumption on the distribution of health (19). For instance, based on certain aggregate measures of health such as mortality or incidence of disease, one can say that the health of the poor in a society is worse off than the non-poor and try to establish causal factors for these differences to inform interventions. Such ordinal comparison of health states can be extended to capture the size of the difference which would allow for comparison of differences (and difference of differences), that is, the measurement of inter-group health differentials on a cardinal scale.

Inter-group differences in health among socially constructed entities such as race and ethnicity are central to the definition of health inequities in most contexts (1). In all racialized social systems, the race placed in the superior position tends to receive greater benefits of the social determinants of health (24); and such placement inevitably leads to health inequities. This is because inequalities resulting from such social stratification are mostly avoidable and amenable to intervention (1, 9).

The conceptualization of health inequities on the basis of inter-group differences is intuitive and often less criticized except perhaps for the fact that it may obscure the attribution of causality (19). This argument is however not peculiar to inter-group differences since making a causal inference with respect to inter-individual differences would also require the aggregation of health differentials at some group level. Other criticisms of inter-group differences revolve around how the groups are defined. While most commonly used population groupings such as sex and socioeconomic status have clear hierarchical orderings, the same cannot be said for other groupings such as occupational classes (25). The informational bases of the health measure are also a concern with inter-group differences, that is, from what perspective are the differences measured: those of the members of a certain group (internal) or that of the researcher (external). When self-reported health measures are used, they present a subjective view which further compounds the comparability of health inequities across time and place (19).

## Health inequities: between utilitarian and egalitarian perspectives

Measuring health inequities involves quantifying those health inequalities that are of moral concerns or unfair due to disparities in the underlying social determinants of health (19). For purposes of the present article, and in order to avoid protracted debate about what constitutes ‘fairness’ (26), discussion on the philosophical perspectives that have been used to explain health inequities will be restricted only to those that may be relevant for evaluating the common measures of health inequities in program evaluation. Prominent among these perspectives are utilitarianism and egalitarianism. The utilitarian perspective proposes that the maximization of aggregate health is more important than the distribution of health within a population (27). At the extreme of utilitarianism, the allocation of benefits to the well-off group at the expense of the disadvantaged group is justifiable under the presumption that those in the well-off group have more capacity to put the benefits to better use and maximize the aggregate outcome for which the benefits were intended (27, 28). The egalitarian perspective on the other hand espouses that ensuring equality in the distribution of an outcome or factor is more important than increasing its aggregate level and it is only when distribution is optimal that its aggregate level truly increases (27). The central issue in egalitarianism is that of equality of humans in terms of worth and value without any special recourse to their socioeconomic position (29). At the extreme of egalitarianism, all groups (including the least-off group) could fair worse, so far as equality is achieved (30).

While both the egalitarian and utilitarian perspectives have been useful in unraveling the notion of fairness in health; perhaps, the most important view that has shaped the definition of health inequities is anchored in Rawls’ theory of social justice (8, 10, 31). The theory refines the egalitarian principles by defining social justice as the optimization of social, political, and economic processes within a society such that the worse-off group is not put at a further disadvantage while favoring the better-off group (22, 32). Rawl's theory of social justice adopts an ‘original position’, a hypothetical locus where individuals are ignorant of their place in society and of the claim they have to social goods (22, 32). From this standpoint, Rawl's suggested that individuals are likely to be fair in a social contract and would act according to two main principles: *the first principle of justice*, which entrenches the equal right individuals have to basic liberties; including the freedom of speech and assembly, political, and personal liberties (22, 32). *The second principle of justice*, which is embodied in the *difference principle*, proposes that individuals within a society, operating from the ‘original position’, would be willing to accept inequalities in the distribution of social and economic advantages so far as it disproportionately benefits the worse-off group (22, 32).

The second principle also encompasses the *equality of opportunity* which suggests that individuals should get what they deserve by merit (and not by any other metric including social status) and that the opportunity to acquire the knowledge and training by which the merit is assessed is open to all (22, 31, 32, 33). Thus, Rawl's second principle attempts to reconcile what is fair under both classical egalitarianism (i.e. an unequal distribution of social advantages to favor the worse-off group) and utilitarianism (that is, a merit-based ascension, which maximizes aggregate outcome, modified by an equal chance to qualify as being meritable within societies).

In propounding the difference principle, Rawls (22) explained that there is a linkage between social processes and inequalities within societies. Hence, some authors have suggested that in order to address health inequities, policies interventions should be formulated to reorder the distribution of social determinants of health (5, 31).

## Operationalizing the definition of health inequities for public health program evaluation

Public health programs as used in this paper are measures or strategies applied to prevent disease, promote health, and prolong life among a population as a whole. Programs designed to address health inequities are often targeted at only the disadvantaged groups to improve one or more dimensions of health access exclusively among this group (34). Examples of such programs include interventions and strategies for lowering financial barriers for the poor, improving health provision for the poor and increasing access to health determinants such as improved water, sanitation, and home environment. The implicit theory is that targeting efforts to improve health access for the disadvantaged group will disproportionately improve health outcomes for this group thereby shifting the distribution of health outcomes for the entire population and narrowing the inequity gap (34, 35).

Therefore, a common approach for operationalizing the definition of health inequities for public health program evaluation involves demonstrating changes in a health outcome within the disadvantaged population only (35, 36). For example, in post-Apartheid South Africa, a pension program was designed to provide a safety net and reduce financial barriers for those of retirement age with insufficient employment-based pension (37). Whereas the program was implemented for the entire population, the impact was assessed only on household members of elderly black/colored adults and not on household members of elderly white adults (37). The results showed that height for age of children who live in black/colored households with a pensioner was significantly higher than those children in similar households without a pensioner (37). This improvement among black/colored children was suggested as a positive effect of the program on narrowing the inequity gap in South Africa (37, 38).

The range is another common approach for operationalizing measures of health inequities in public health program evaluation. This quantifies the absolute or relative difference in a health measure between disadvantaged and advantaged groups (25). For example, a program in Afghanistan contracted the delivery of basic health services to non-governmental organizations in the rural areas to improve health access for the poor (39). The assessment of the program compared the odds of utilization of services under different contracting arrangements between poor and non-poor (39). The results showed that the odds ratio of the poor relative to the non-poor seeking care at health facilities under one of the contracting arrangements was significantly higher compared to those exposed to non-contracted health facilities (39). Based on the analysis, the conclusion was that the contracting arrangement was effective in reducing health inequity in Afghanistan (39).

Other measures have operationalized health inequities as a composite indicator that reflects distribution of health outcome between the poor and non-poor in a given population. Most of these measures have their origin in economics and were designed primarily to capture the socioeconomic inequalities in health (25, 40). These composite indicators include the Lorenz curve and Gini coefficient, pseudo-Lorenz curve and pseudo-Gini coefficient, concentration curve and index, and the slope of relative indices of inequality (41). These indicators are operationalized to quantify health inequities by plotting population of individuals or groups ranked by their socioeconomic position or health status against the cumulative proportions of a health measure (41). For example, the impact of conditional cash transfer programs targeted at reducing financial barriers and improving health status of the poor in Brazil and Mexico was assessed using the Gini coefficient in one study (42). The results showed that the Gini index in Brazil and Mexico fell by 4.7 and 5%, respectively between 1995–1996 and 2004 and that the conditional cash transfer program accounted for 21% of this fall in both countries (42). The decline in the Gini index, which ranges from 0 (perfect equality) to 1 (perfect inequality), suggested that the program has contributed to narrowing the inequity gap in the two countries.

Other operational measures useful for assessing impact of public health programs on health inequities include the index of dissimilarity and regression techniques, (25, 41) and Table 1 provides a brief description of these measures.

**Table 1:** Common approaches for operationalizing health inequities in program evaluation

Measure of health inequity	Description	Indicator of inter-group or inter-individual inequalities	Indicator of health inequalities in relation to socioeconomic position	Able to reflect the difference principle (sensitive to the absolute transfer of measures of health or income/wealth between groups)	Indicator of health inequalities in relation to other population groupings of interest (race, sex and geographical location)	Example papers
Before and after measures within only the disadvantaged population	Comparison (**difference or ratio**) of the measure of health within only one group to demonstrate impact of a program on the measure of health	None	No	Does not reflect transfer of either health or income/wealth	No	Case (2001). Does money protect health status? Evidence from South African pensions (37)
Range	Comparison (**difference or ratio**) of the measure of health between population groups. The comparison group could be any of the groups. Also, include **shortfalls** which compare measure for each group to a norm before inter-group comparison.	Can be operationalized for both inter-group and inter-individual differences. It takes no account of the sizes of the groups being compared when used as a measure of inter-group difference	Yes	Could be used to reflect transfer of both health and income/wealth	Yes	Townsend and Davidson (1982). Inequalities in health: The Black report (43)
Lorenz curve and Gini coefficient	The Lorenz curve is the plot of cumulative proportions of the population ranked by health (from the sickest person to the healthiest) against the cumulative proportion of health. The Gini coefficient is twice the area between the Lorenz curve and the diagonal. It ranges from 0 to 1 (i.e. from complete equality to when all the health is concentrated in the hands of one person).	Inter-individual indicator	No	Does not reflect transfer of either health or income/wealth	Yes (when combined with stratified analysis^a^)	Le Grand and Rabin (1986). Trends in British health inequality (15)
Pseudo-Lorenz Curve and pseudo-Gini coefficient	Plot of cumulative proportions of population grouped by social class and ranked by their mean health status (from the group with lowest mean health status to the group with the highest mean health status) against the cumulative proportion of health. The pseudo-Gini coefficient is twice the area between the pseudo-Lorenz curve and the diagonal. It ranges from 0 to 1 (i.e. from complete equality to when all the health is concentrated in the hands of one group)	Inter-group indicator. It accounts for the population sizes of the groups being compared	No	Could be used to reflect transfer of health only	Yes	Leclerc, Lert and Fabien (1990). Differential mortality: some comparison between England and Wales, Finland and France (44)
Index of dissimilarity	The sum of the absolute difference of share of total population health and share of population for each group divided by two.	Inter-group indicator. It accounts for the population sizes of the groups being compared	No	Could be used to reflect transfer of health only	Yes	Pappas (1993). The increasing disparity in mortality between socioeconomic groups in the United States, 1960 and 1986 (45)
Concentration curve and index	The concentration curve plots the cumulative proportions of the population ranked by their socioeconomic position (beginning with the most disadvantaged to the least disadvantaged) against the cumulative proportions of health. The concentration index (ranges from −1 to +1; i.e. from health being concentrated in the hands of the most disadvantaged to the least disadvantaged) is twice the area between the concentration curve and the diagonal.	Can be operationalized for both inter-group and inter-individual differences. It accounts for the population sizes of the groups being compared when used as a measure of inter-group. difference	Yes	Could be used to reflect transfer of health. It does not typically reflect the transfer of income/wealth unless there is a related change in the income/wealth ranking	No	Wagstaff, Paci and van Doorslaer (1991). On the measurement of inequalities in health (41)
Slope and relative indices of inequality (SII and RII)	SII is the slope of the regression line of the mean health status for each group against the relative rank of socioeconomic status (not health status) beginning with the most disadvantaged. It assumes a linear relationship between the two properties. Both properties are weighted by the square root of the group size. RII is SII divided by the mean health status for the entire population.	Inter-group indicator. It accounts for the population sizes of the groups being compared	Yes	Could be used to reflect transfer of health. It does not reflect the transfer of income/wealth unless there is a related change in the income/wealth ranking	No	Mackenbach et al. (2008). Socioeconomic Inequalities in health in 22 European countries (46)
Other regression techniques	Regression of the health status measure against the measure that defines the population groupings (e.g. socioeconomic status). Assumes a linear relationship between the two properties.	Inter-group indicator. It accounts for the population sizes of the groups being compared when weighted least square regression is used and weights are defined by group size	Yes	Could be used to reflect transfer of both health and income/wealth	Yes	Valkonen (1989). Adult mortality and level of education: a comparison of six countries (47)

a Stratified analysis in this case implies computing Gini coefficient of health within each strata of the population grouping.

## Theoretical assessment of common operational measures of health inequities

In Table 1, common measures of health inequities are assessed against four criteria to understand the extent to which each incorporates important dimensions for defining health inequities. These criteria are based on the theoretical analyses outlined in the earlier section and on axioms of an ideal health inequity indicator from a review of literature (48, 49). As was discussed earlier with the Black et al. (14) and Le Grand and Rabin (15) examples, conclusions about changes in the inequity gap could differ depending on whether the measure captures inter-individual or inter-group differences. A robust measure of health inequity should ideally capture both inter-individual and inter-group differences to forestall any erroneous conclusion; this is one of the four criteria assessed (48).

Also, because most public health programs designed to address health inequity are targeted at shifting the distribution of health and its determinants in favor of the disadvantaged group (Rawl's difference principle) (22, 48), a robust measure of health inequity should distinguish transfer of benefits over time from an advantaged group or individual to a disadvantaged group or individual from any reverse transfer (i.e. from disadvantaged to advantaged). This criterion is assessed as whether the measure could reflect and distinguish the absolute transfer of health benefits and change in position between groups or individuals.

Whereas socioeconomic inequalities in health forms the basis of the definition of health inequities in most societies (6, 7), health inequity could be observed among groups or individuals defined by other social and biological characteristics. Hence, two additional criteria examined whether the indicator could reflect socioeconomic inequalities in health as well as capture inequalities in relation to other population groupings that could form the basis of inequities for example, race, sex, and geographical location.

Summarily, the four criteria assessed are whether the inequity measure captures either inter-group or inter-individual differences or both; reflects health differences in relation to socioeconomic position; is sensitive to the absolute transfer of measures of health or income/wealth between groups; and able to capture health differences in relation to other population groupings. It is interesting to note that no single measure satisfies all the criteria assessed apart from the range operationalized either directly or with regression techniques.

Based on the definition of health inequities outlined above, inequity is inherently comparative irrespective of the informational bases from where it is assessed. Hence, assessing changes in measures of health inequity within only the disadvantaged group may be insufficient since they fail to capture what is happening concomitantly among the advantaged group. In the South Africa pension program example (37), it is in fact possible that the health improvements observed comparing children living in black households with or without a pensioner similarly occur in white households with or without a pensioner but to a greater degree. That is, while the program benefits all; white households disproportionately capture more of the benefits thereby widening the inequity gap contrary to the conclusion of the study.

For composite indicators such as the concentration index, while it can show the transfer of health between groups to show if a program has narrowed or increased the inequity gap, it cannot show the transfer of wealth/income between groups which could also be a reason for changes in the inequity gap. This is because it only accounts for the rank of wealth/income and not for changes in the levels of wealth/income between groups (50). Also, very few of the measures assessed can be extended to quantify health inequities among other population groupings apart from socioeconomic groups. For this reason, the use of some of these measures may be limited for examining health inequities in its various dimensions.

The composite indicators also require strong assumptions that the health measure is normally distributed and measured on a ratio scale with non-negative value like income (20, 51) These assumptions may not necessarily hold for various measures of health however. Therefore, some have cautioned that the application of these methods to health variables that are not measured on a ratio scale (such as categorical self-reported health outcomes) may be an incorrect approach (51). Also, some of these measures become invariant at the extremes of health coverage (51). For example, the concentration index which ranges from −1 to +1, that is from a health distribution that maximally favors the poor to that which favors the non-poor, would equal to or be close to zero when health coverage is very low or high (44). Despite these drawbacks, the income-inequality-related measures are useful for operationalizing health inequities that are based on socioeconomic health inequalities and comparing trend of health for different populations when applied under the correct assumptions.

The range continues to be a versatile measure of health inequities because it is simple to interpret and does not require any strong assumption about the distribution of the health measure or its measurement scale (24). Moreover, the range can be readily combined with various regression techniques to explore causal models of health inequities; which is why it is particularly relevant for assessing health inequities in program evaluation. Also, programs implemented at a small scale can be assessed with the range without rigorous assumptions about sample distribution using non-parametric statistical approach.

The range is not without its flaws. It has been pointed out that the range does omit intermediate groups when the number of groups exceeds two; (41) unless these groups are re-grouped into two broad classes. Such re-grouping however, may not have any sound conceptual basis, in which case, its operationalization might completely alter the evaluation question of interest. Also, the range does not account for sample size of the groups, that is, it does not incorporate information on the actual number of people within the population in a particular group and only captures differences in outcome between groups which may not be relevant for policy given the size of the population affected (18). This drawback may however disappear if the program is rigorously designed for causal inference and sufficiently powered to test differences or ratio between population groups.

Indeed, each measure of health inequities quantifies a different perspective of inequity. It is therefore imperative to clearly understand the limit of each and how it operationalizes the definition of health inequities. Whereas the decision on which measure to use for program evaluation should be based on how the program is conceptualized to impact health inequities in a particular context, it may be advantageous however to use more than one measure including the range for a robust assessment of health inequities.

## Summary

The recent global call for universal coverage for health has generated renewed interest in public health programs for narrowing inequity gaps and approaches for quantifying these changes in program evaluation. The definition and approaches for quantifying health inequities require strong conceptual basis. This paper reviewed the various dimensions necessary for defining health inequities. To examine the extent to which different measures of health inequities incorporate these conceptual underpinnings, four main criteria were identified and used to assess the different measures. These factors are whether the measure: is an indicator of inter-group or inter-individual health inequalities; reflects health inequalities in relation to socioeconomic position; reflects the difference principle, that is how sensitive is the measure to the absolute transfer of health or income/wealth between groups; and finally, if it could be used to capture inequalities in relation to other population groupings. None of these measures satisfied all the four criteria, except the range. Whereas each measure quantifies some perspectives of health inequities, using a measure within only the disadvantaged population does not measure health inequities in a meaningful way, even using before and after changes. For a more complete assessment of how programs affect health inequities, it may be useful to use more than one measure.

## Footnotes

1 The concept of health disparity has sometimes been extended not only to describe health inequalities broadly but also to qualify those health inequalities that are linked to unacceptable differences in social determinants of health (1).
